# Profiling of Intracellular Metabolites: An Approach to Understanding the Characteristic Physiology of *Mycobacterium leprae*

**DOI:** 10.1371/journal.pntd.0004881

**Published:** 2016-08-01

**Authors:** Yuji Miyamoto, Tetsu Mukai, Masanori Matsuoka, Masanori Kai, Yumi Maeda, Masahiko Makino

**Affiliations:** Department of Mycobacteriology, Leprosy Research Center, National Institute of Infectious Diseases, Higashimurayama, Tokyo, Japan; Fondation Raoul Follereau, FRANCE

## Abstract

*Mycobacterium leprae* is the causative agent of leprosy and also known to possess unique features such as inability to proliferate *in vitro*. Among the cellular components of *M*. *leprae*, various glycolipids present on the cell envelope are well characterized and some of them are identified to be pathogenic factors responsible for intracellular survival in host cells, while other intracellular metabolites, assumed to be associated with basic physiological feature, remain largely unknown. In the present study, to elucidate the comprehensive profile of intracellular metabolites, we performed the capillary electrophoresis-mass spectrometry (CE-MS) analysis on *M*. *leprae* and compared to that of *M*. *bovis* BCG. Interestingly, comparison of these two profiles showed that, in *M*. *leprae*, amino acids and their derivatives are significantly accumulated, but most of intermediates related to central carbon metabolism markedly decreased, implying that *M*. *leprae* possess unique metabolic features. The present study is the first report demonstrating the unique profiles of *M*. *leprae* metabolites and these insights might contribute to understanding undefined metabolism of *M*. *leprae* as well as pathogenic characteristics related to the manifestation of the disease.

## Author Summary

*Mycobacterium leprae*, the causative agent of leprosy, has unique physiological features including being uncultivable in artificial media. This fact raises the possibility that *M*. *leprae* possesses specific metabolism that are different from other cultivable mycobacteria. Among the components of *M*. *leprae*, the glycolipids are known to be involved in pathogenicity, while the dynamics of intracellular metabolites such as organic acids, amino acids and nucleic acids remain unclear. Aiming to understand the metabolism of *M*. *leprae*, we characterized the profile of intracellular metabolites. Unexpectedly, we found that amino acid species are significantly accumulated, while most of intermediates related to central carbon metabolism markedly decreased in the metabolite fraction of *M*. *leprae*, as compared with that of other mycobacteria. These specific metabolic features of *M*. *leprae* was presented for the first time and these insights may contribute to understanding the mechanism of physiology including obligate growth *in vivo*, which is one of the key characteristics of leprosy.

## Introduction

*Mycobacterium leprae*, the causative agent of leprosy, is an obligate intracellular pathogen having unique features. The doubling time of *M*. *leprae* is about 14 days as compared to approximately 24 hours of *M*. *tuberculosis* or the vaccine strain *M*. *bovis* BCG. The inability to cultivate *M*. *leprae in vitro* may be due to its intrinsic characteristics of inert nature, probably resulting from the reduced coding capacity of *M*. *leprae* genome. Comparative genomics have shown that there are numerous pseudogenes (1,116 pseudogenes versus 1,604 functional genes) in *M*. *leprae* [1]. So far key studies including comparative genomics and proteomics have been performed, and its unique characteristics have been elucidated [1–4]. However, the interaction of the changes in genomic structures or protein expressions leading to the uncultivable nature of *M*. *leprae* is still unclear. On the other hand, the cell envelope components known to be highly involved in the differentiation of mycobacterial species are becoming clearer. Generally, various glycolipids and lipids, which are abundantly present in the outer layer of mycobacterial cell envelope, play an important role in achieving the pathogenicity including resistance to immune response and entrance into host cells [5]. *M*. *leprae* also has most of such glycolipids in common [6], while the phenolic glycolipid-I (PGL-I) is shown to appear specifically in *M*. *leprae* as a one of major cellular components involved in pathogenicity [7–8].

Focusing on the cytosol of mycobacteria, various metabolites such as free amino acids or organic acid are present, which could be the valuable metabolic fuels, for subsequent intermediary metabolism. Therefore, understanding of the intricate balance of these metabolites, which play a role in driving and maintaining the cellular metabolism, is important. In cultivable organisms like *M*. *tuberculosis*, their dynamics has been investigated under different growth conditions and its alterations have been shown to be associated with the physiological feature as well as unique life cycle [9–12]. However, in the case of *M*. *leprae*, there have been no studies focusing on the fate of intracellular metabolites, which are assumed to be involved in the cellular metabolism. In some studies, the detection of intermediates was performed by labeling with isotopes or in other studies, enzymatic activity was measured by biochemical means [13–16]. Wheeler et al. showed that in *M*. *leprae* carbon from glycerol could be incorporated into the glycerol moiety of acylglycerols but not into the fatty acid moieties [16]. Unfortunately, most of other pathways and metabolism remain unknown probably due to the difficulties in culturing the bacilli. In addition, although the genes involved in basic metabolism are conserved without large deletions, sporadic distribution of pseudogenes was observed in *M*. *leprae* genome, [1], which make the speculation of the metabolism from the genomic analyses of *M*. *leprae* difficult. These facts suggested that genomic analysis alone is not sufficient for elucidating whole metabolisms associated with its unique physiology.

Recently, metabolomics approach using sera of the leprosy patients was undertaken. Significant increase of certain polyunsaturated fatty acids and phospholipids in high bacterial index patients were observed [17]. Also urinary metabolites could discriminate endemic controls from untreated patients, as well as leprosy patients with reversal reactions [18].

In this study, we focused on characterizing the quantitative and qualitative profile of intracellular metabolites in *M*. *leprae* by capillary electrophoresis-mass spectrometry (CE-MS) analysis, and compared them with those in *Mycobacterium bovis* BCG, which would consequently lead to the elucidation of pathogenic mechanisms of leprosy.

## Methods

### Mycobacterial culture and metabolite preparation

*M*. *leprae* Thai-53 strain was infected into the footpads of each nude mouse (BALB/c nu/nu) [19]. To propagate *M*. *leprae*, infected nude mice were maintained for 12 months in an isolated chamber. In order to exclude the possibility of getting wrong results, from the mouse-derived metabolites in *M*. *leprae* extract, we also prepared the uninfected nude mice which were maintained for the same period as *M*. *leprae*-infected nude mouse. Briefly, footpads dissected from *M*. *leprae*-infected and uninfected nude mouse were mechanically homogenized in Hanks' balanced salt solution (HBSS) as previously described [20, 21]. An aliquot containing 2.5×10^10^ bacilli was taken from footpad homogenate of *M*. *leprae-*infected nude mouse and suspended in 5 ml of HBSS. In case of footpad homogenate from uninfected nude mouse, the volume equivalent to an average of above 2.5×10^10^ bacilli-contained aliquot was also taken and suspended in 5 ml of HBSS. For comparative study of the metabolites of *M*. *leprae*, we used *M*. *bovis* BCG Tokyo strain as control mycobacteria. 2.5×10^10^ bacilli were harvested from 1 week-culture in Middlebrook 7H9 broth supplemented with 10% ADC enrichment and suspended in 5 ml of HBSS. Above 5ml-HBSS suspensions containing 2.5×10^10^ mycobacterial cells and uninfected footpad homogenate were then incubated with 0.05% trypsin at 37°C for 1 hour. According to the metabolite extraction procedures [22–24], trypsin-treated samples were collected by suction filtration using the Isopore Membrane Filter (HTTP04700) (Millipore, Massachusetts, USA). The collected samples were washed with Milli-Q water, and then exposed to methanol with Internal Standard Solution 1 (Human Metabolome Technologies, Yamagata, Japan) to obtain crude intracellular extracts. These were further treated with chloroform to remove the lipid components, and then filtrated with 5-kDa cut-off filter (UFC3LCCNB) (Human Metabolome Technologies, Yamagata, Japan) to yield the intracellular metabolite extract suitable for CE-MS analysis. In three groups, all procedures were independently performed in triplicate.

### CE-MS and statistical analysis

For metabolite identification and quantification, Agilent CE-TOFMS System was basically performed on above prepared extracts, according to the conditions previously reported [22–24]. Capillary electrophoresis was carried out with a fused silica capillary whose diameter and length are 50 μm and 80 cm, respectively. Cationic and anionic metabolites were ionized in the positive ion mode (4 kV) and negative ion mode (3.5 kV), respectively. The data were processed using MasterHands ver.2.16.0.15 (Keio University) for retrieving the *m/z* value, migration time and peak area. Each metabolite was identified from its *m/z* value and migration time by searching against metabolite database (Human Metabolome Technologies, Yamagata, Japan). Relative quantity of each metabolite was estimated by comparison of peak area with that of a standard compound in Internal Standard Solution 1 and resultant values were determined as relative peak area. In data processing of relative peak area, only the metabolites detected in triplicate were used for calculation of means, standard deviations and other analyses. By Welch’s t test, we assessed whether relative quantity of each metabolite was statistically different between two groups. Principal component analysis (PCA) was performed by using the MetaboAnalyst 3.0 (University of Alberta and McGill University) [25].

### Ethics statement

Animal experiments were carried out in strict accordance to "Act on Welfare and Management of Animals" enacted in 1973. The protocol was approved by the Experimental Animal Committee of the National Institute of Infectious Diseases, Tokyo (Permit Number: 214001), whose guidelines are established by the Ministry of Health, Labour and Welfare, Japan (MHLW).

## Results and Discussion

Since *M*. *leprae* cannot be cultivated *in vitro*, the propagation in experimental animals such as nude mice and armadillo is currently the only way to obtain sufficient *M*. *leprae* for biochemical experiments. In this study, we propagated *M*. *leprae* in footpads of nude mice for 12 months. Footpads from *M*. *leprae*-infected and uninfected nude mice were processed in similar manner as described in the Methods, and the resultant extracts were analyzed.

**Fig 1:**
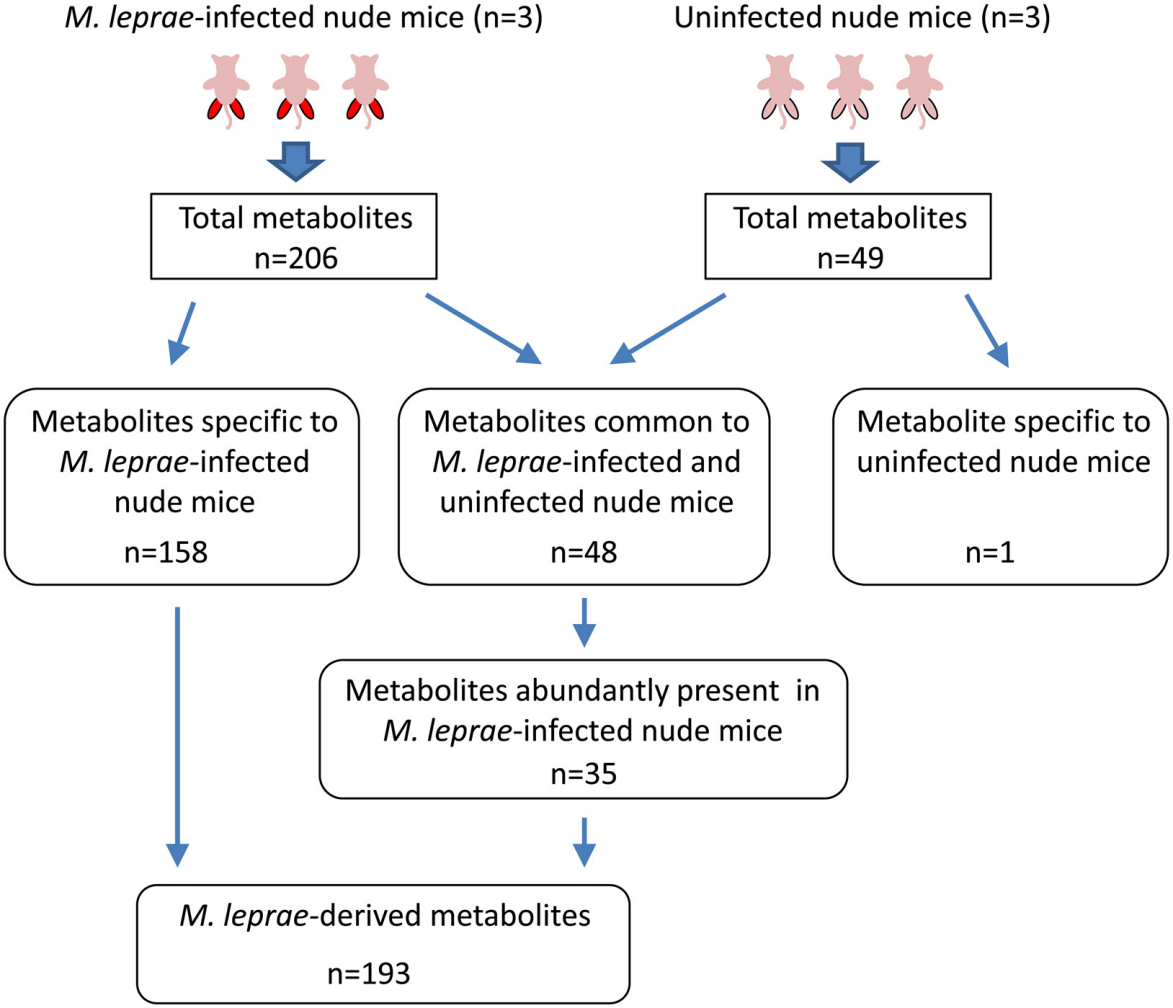
Schematic representation for retrieving the *M*. *leprae*-derived metabolites identified by CE-MS analysis. In each of common 48 metabolites, the mean of triplicate values (relative peak areas) of uninfected nude mice was subtracted from all triplicate value of *M*. *leprae*-infected nude mice and resultant 35 metabolites were retrieved as abundantly present in *M*. *leprae*-infected nude mice.

As shown in Fig 1, CE-MS analysis showed that 206 and 49 metabolites were present in detectable amount in the extracts of footpads dissected from *M*. *leprae*-infected (S1 Table) and uninfected nude mice (S2 Table), respectively (n = 3). Among 206 metabolites, 158 metabolites were specifically observed in *M*. *leprae*-infected nude mice, while only one compound was detected in uninfected nude mice (Fig 1). These observations mean that more than 75% of the detected metabolites in *M*. *leprae*-infected nude mice are derived from *M*. *leprae* itself. On the other hand, 48 metabolites from *M*. *leprae*-infected nude mice were also found in the extracts of uninfected nude mice. Quantitative comparison of 48 compounds between *M*. *leprae*-infected and uninfected nude mice revealed that most of relative peak areas from uninfected nude mice are much lower than those from *M*. *leprae*-infected nude mice (S1 Fig). To acquire the authentic value of the metabolites derived from *M*. *leprae*, we used the background subtraction method. Mean value of each metabolite obtained from uninfected nude mice was subtracted from all triplicate value of *M*. *leprae*-infected nude mice and thereby retrieved 35 metabolites which were abundantly present in *M*. *leprae*-infected nude mice (Fig 1). Eventually, 193 metabolites were totally determined to be specifically present in the *M*. *leprae* extract (S3 Table) (Fig 1).

Although it became apparent that *M*. *leprae* possessed 193 specific intracellular metabolites, it is unknown how their qualitative and quantitative profiles differ from other mycobacteria. *M*. *leprae* is known to have unique properties such as long-term obligate growth *in vivo*, which is quite different from other mycobacteria, indicating that it is difficult to select the control mycobacteria for comparison. In this study, for comparative profiling of the *M*. *leprae* metabolites, we chose *in vitro*-cultured *M*. *bovis* BCG. Unlike *M*. *leprae*, *M*. *bovis* BCG does not remain in the footpad where inoculated but get disseminated all over the body and sufficient bacilli cannot be retrieved for analyses. [26]. By performing the extraction procedures of *in vitro*-cultured *M*. *bovis* BCG under the same conditions as *M*. *leprae*, we obtained the metabolite extracts of *M*. *bovis* BCG for comparative study.

**Fig 2:**
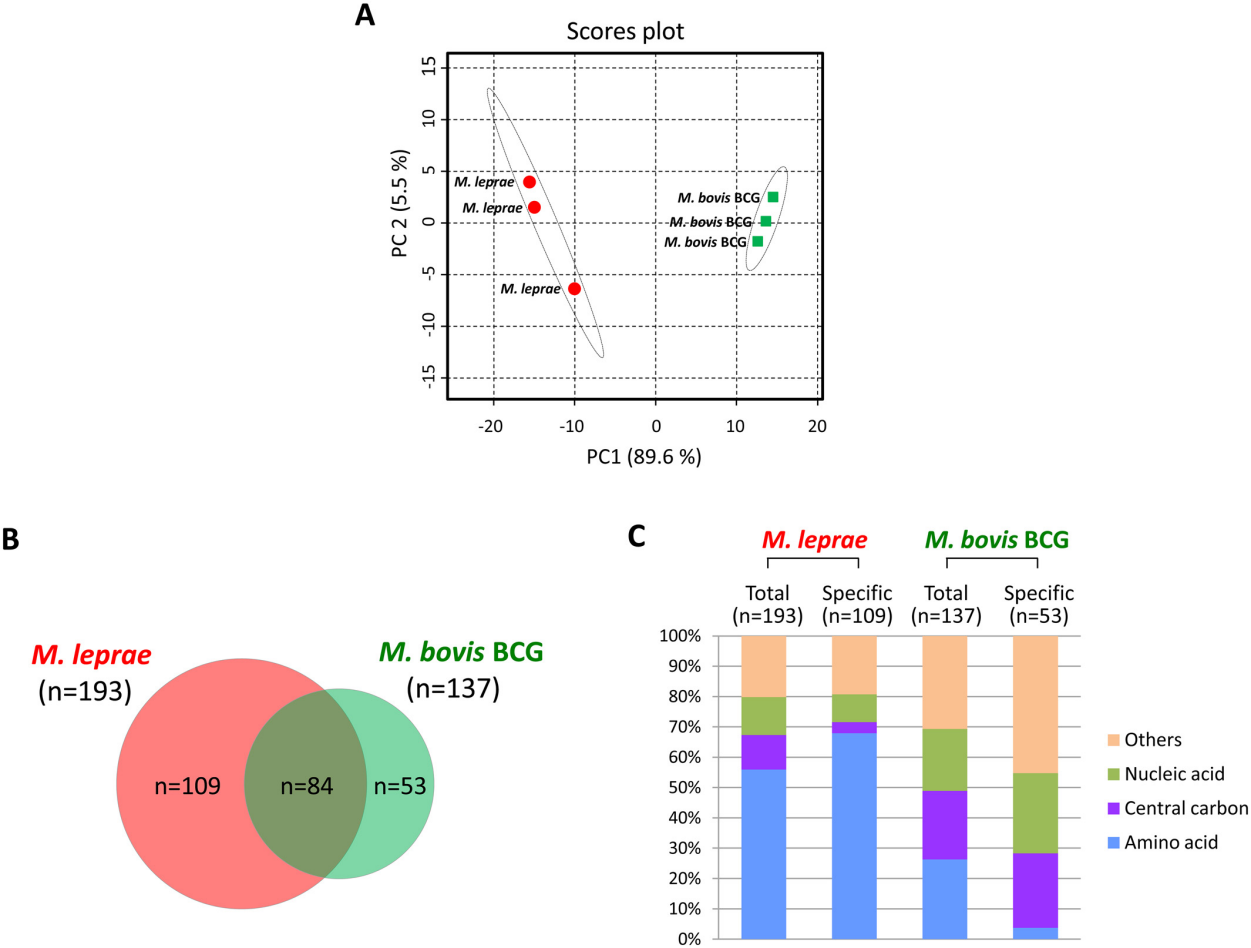
Comparative profiling of metabolites detected from *M*. *leprae* and *M*. *bovis* BCG. A) Principal component analysis (PCA) on relative peak area of metabolites detected from three independent groups of *M*. *leprae* and *M*. *bovis* BCG. Total relative peak area for analysis was derived from 193 metabolites of *M*. *leprae* and 137 metabolites of *M*. *bovis* BCG. B) Venn diagram of identified metabolites. C) Proportion of the classified metabolites based on their putative functions in each metabolism. Each of mycobacterial metabolites was derived from 2.5x10^10^ cells.

CE-MS analysis of *M*. *bovis* BCG indicated that 137 compounds could be detected (S4 Table). To obtain a comprehensive profile of metabolites, we performed the principal component analysis (PCA) on relative peak area of metabolites detected from three independent groups of *M*. *leprae* and *M*. *bovis* BCG (Fig 2A). The results showed that the groups cluster within each species but their clusters are clearly separated, suggesting that the sort and quantity of detected metabolites from *M*. *leprae* are close within three analyzed groups, but they are quite distinguishable from those of *M*. *bovis* BCG groups whose profiles were also similar to each other (Fig 2A). Therefore, this implied that metabolic profile of *M*. *leprae* and *M*. *bovis* BCG are functionally distinct. Comparison of detected metabolites between two species indicated that 84 metabolites are in common, while 109 and 53 metabolites are specifically observed in *M*. *leprae* and *M*. *bovis* BCG, respectively (Fig 2B). Additionally, we assessed and classified the metabolites in terms of their function in each category of metabolism (Fig 2C). As a consequence, we found that 56% of the total metabolites of *M*. *leprae* were categorized under the amino acid metabolism group, while in *M*. *bovis* BCG only 26% of the total metabolites belonged to the amino acid metabolism (Fig 2C). On the other hand, as for the metabolites associated with central carbon and nucleic acid metabolism, it was observed that their proportion in *M*. *bovis* BCG was almost twice as much as the metabolites of *M*. *leprae* (central carbon: 23% vs. 11%; nucleic acid: 20% vs. 12% in *M*. *bovis* BCG vs *M*. *leprae* respectively)(Fig 2C). These differences were more pronounced when the population of specifically detected metabolites from *M*. *leprae* and *M*. *bovis* BCG were considered. The amino acid-related compounds constitute 68% of 109 metabolites, which were specifically observed in *M*. *leprae*, but its proportion in 53 *M*. *bovis* BCG-specific metabolites was only 4% (Fig 2C). On the contrary, the percentage of the compounds related to the central carbon and nucleic acid metabolism was shown to be quite high in *M*. *bovis* BCG-specific metabolites, compared to those in *M*. *leprae-*specific metabolites (central carbon: 25% vs. 4%; nucleic acid: 26% vs. 9% in *M*. *bovis* BCG vs *M*. *leprae* respectively)(Fig 2C). These results indicated that, in *M*. *leprae*, amino acid-related compounds constituted a larger proportion of detected metabolites, while detection of metabolites associated with central carbon and nucleic acids were relatively small, compared to those of *M*. *bovis* BCG.

**Fig 3:**
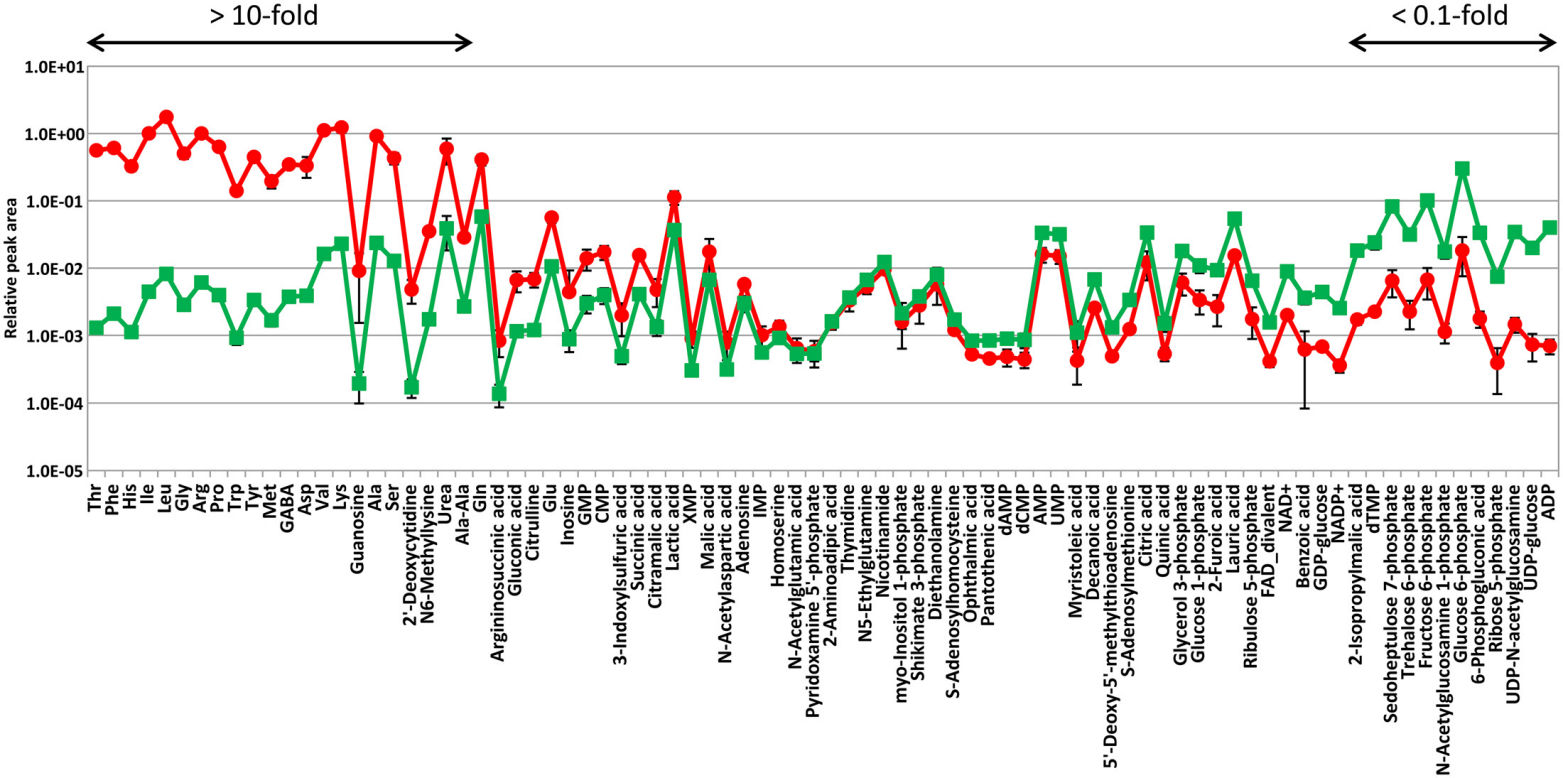
Quantitative comparison of 84 metabolites common to *M*. *leprae* and *M*. *bovis* BCG. Relative peak areas are expressed as means± S.D. of triplicate. These values were retrieved by CE-MS analysis performing on methanol extract from 2.5x10^10^ cells of each mycobacteria collected by filtration. As for those of *M*. *leprae*, nude mice-derived values were subtracted. Red circle and green square represent the mean value of each metabolite detected from *M*. *leprae* and *M*. *bovis* BCG, respectively. 84 metabolites are arranged in descending order of their mean ratio (*M*. *leprae*/*M*. *bovis* BCG). Of these metabolites, their mean ratio showing over 10-fold and less than 0.1-fold are indicated by arrows.

To better explore the mechanisms governing the fate of *M*. *leprae*, we focused on the quantitative evaluation of 84 metabolites, which were common to both *M*. *leprae* and *M*. *bovis* BCG (S5 Table). When the mean ratio of each relative peak area (*M*. *leprae*/*M*. *bovis* BCG) was arranged in decreasing order, it was found that 26% (22/84) of the metabolites significantly showed large differences (>10-fold), while 14% (12/84) showed <0.1-fold differences (Fig 3). Additionally, functional classification of the metabolites having 10-fold difference in the mean ratio revealed that most of the compounds listed are involved in amino acid metabolism (Table 1). This is supported by the fact that *M*. *leprae*-specific metabolites were dominated by those related to amino acid metabolism (Fig 2C), suggesting that amino acid and its derivatives abundantly accumulated as intracellular metabolites in *M*. *leprae*, when compared to those metabolites of *M*. *bovis* BCG. These results raise two possibilities regarding the metabolic aspects of *M*. *leprae*: (1) *M*. *leprae* itself has the capacity to produce high amount of amino acids, (2) *M*. *leprae* activates the uptake of amino acids from host, in order to maintain and control the metabolism suited for long-term, obligate growth *in vivo*. At present, it is not clear which mechanism could better explain the cause of amino acid accumulation, because no direct evidence was obtained from the *M*. *leprae* genomic analyses of the regions that could be involved. Probably there is yet unknown mechanism which causes the amino acid accumulation. On the contrary, Table 2 show that the metabolites in the cluster showing less than 0.1-fold differences in their mean ratio of relative peak area (*M*. *leprae*/*M*. *bovis* BCG) dominantly belonged to the intermediates related to the central carbon metabolism, for example metabolites such as glucose-6-phosphate, fructose-6-phosphate, sedoheptulose 7-phosphate, 6-phosphogluconic acid and ribose 5-phosphate. These metabolites play a critical role in cellular pathways of energy metabolism as well as other basic cellular processes. The same tendency is also observed when metabolites were classified according to their function (Fig 2C), suggesting that the central carbon metabolism in *M*. *leprae* is strikingly declined or repressed compared to those in *M*. *bovis* BCG. These results are exemplified by the *M*. *leprae* genomics demonstrating that around half of genes related to energy metabolism tuned out to be the pseudogenes, which might lead to the functional defect [1]. It is generally hypothesized that lack of energy generation machinery is the main reason for the inability of the bacilli to proliferate *in vitro*, while there are only genomics analysis available to prove the hypothesis. Thus, phenotypically, our study of comparative metabolomics for the first time supported this hypothesis.

**Table 1:** Functional classification of the metabolites having over 10-fold difference in the mean ratio of relative peak areas [*M*. *leprae* (n = 3)/*M*. *bovis* BCG (n = 3)].

Name	Class	Difference of relative peak area	
		Mean ratio	*P*-value
Thr	Amino acid	432.366	0.007
Phe	Amino acid	289.962	0.005
His	Amino acid	289.694	0.008
Ile	Amino acid	224.924	0.005
Leu	Amino acid	214.813	0.004
Gly	Amino acid	176.775	0.01
Arg	Amino acid	163.532	0.005
Pro	Amino acid	159.291	0.003
Trp	Amino acid	152.172	0.003
Tyr	Amino acid	133.469	0.006
Met	Amino acid	116.309	0.016
GABA	Amino acid	92.422	0.002
Asp	Amino acid	85.865	0.038
Val	Amino acid	68.687	0.005
Lys	Amino acid	53.537	0.009
Guanosine	Nucleic acid	47.326	0.178
Ala	Amino acid	38.597	0.006
Ser	Amino acid	33.751	0.013
2'-Deoxycytidine	Nucleic acid	28.377	0.049
*N*^6^-Methyllysine	Amino acid	20.319	0.002
Urea	Amino acid	15.24	0.06
Ala-Ala	Amino acid	10.622	0.001

Metabolites are arranged in descending order of the mean ratio of each relative peak area.

**Table 2:** Functional classification of the metabolites having less than 0.1-fold difference in the mean ratio of relative peak areas [*M*. *leprae* (n = 3)/*M*. *bovis* BCG (n = 3)].

Name	Class	Difference of relative peak area	
		Mean ratio	*P*-value
2-Isopropylmalic acid	Amino acid	0.095	0.0003
dTMP	Nucleic acid	0.094	0.019
Sedoheptulose 7-phosphate	Central carbon	0.079	0.003
Trehalose 6-phosphate	Other	0.071	0.002
Fructose 6-phosphate	Central carbon	0.066	0.007
*N*-Acetylglucosamine 1-phosphate	Central carbon	0.064	0.017
Glucose 6-phosphate	Central carbon	0.061	0.006
6-Phosphogluconic acid	Central carbon	0.053	0.006
Ribose 5-phosphate	Central carbon	0.052	0.011
UDP-*N*-acetylglucosamine	Central carbon	0.043	0.002
UDP-glucose	Central carbon	0.037	0.0001
ADP	Central carbon	0.017	0.006

Metabolites are arranged in descending order of the mean ratio of each relative peak area.

*M*. *tuberculosis* is known to possess the ability to degrade and use cholesterol as an energy source and for the biosynthesis of mycobacterial lipids. In *M*. *leprae*, the presence of host-derived cholesterol plays important role in the process of intracellular survival [27]. However, *M*. *leprae* lost essentially all the genes associated with cholesterol catabolism but retained only the ability to oxidize cholesterol to cholestenone, indicating that cholesterol metabolism was not coupled to central carbon metabolism in *M*. *leprae* [28]. Lipids in the foamy macrophages and Schwann cells were shown to be derived from the host lipids, favoring bacterial survival [29, 30]. In these contexts, *M*. *leprae* has its own unique metabolic pathways to sustain its growth and multiplication, which has to be further elucidated.

Focusing on minute detail of the *M*. *leprae* genome, numerous pseudogenes are distributed in the genomic region of each metabolic pathway, suggesting that such unique features of the genome might be one of the factors influencing the characteristic profiles of intracellular metabolites. However, at present, it is difficult to identify the pseudogenes causing *M*. *leprae*-specific profiles because each pathway is interrelated and is not thoroughly investigated, which leads to complications in deciphering the metabolism in *M*. *leprae*. Therefore, generation of mutants having mutations that mimic the pseudogenes of *M*. *leprae* in cultivable mycobacteria might partly elucidate the relationship between uniqueness in genomic organization and the metabolic profile obtained by CE-MS analysis.

Present study demonstrated that the dynamics of intracellular metabolites in *M*. *leprae* is quite different from those in *M*. *bovis* BCG. Although it is necessary to perform the metabolite profiling on other mycobacteria for more precise evaluation of *M*. *leprae* metabolism, the result retrieved from comparison with *M*. *bovis* BCG would partly contribute to the uncovering the *M*. *leprae* physiology associated with onset of leprosy. In *M*. *tuberculosis*, metabolomics analysis has been performed on *in vitro*-grown cells under conditions such as hypoxia and nutrient starvation which stimulate *in vivo* growth, while no study performing the metabolomics on actually *in vivo-*grown cells was reported [9–12]. Thus, our findings regarding *in vivo*-grown *M*. *leprae* might provide insights into the understanding of not only its physiology but also the metabolic behavior of mycobacteria in host, which remains unresolved.

## Supporting Information

S1 Fig Quantitative comparison of 48 compounds detected commonly in *M*. *leprae*-infected and uninfected nude mouse. Red and blue bar represent the relative peak areas of each metabolite detected from *M*. *leprae*-infected and uninfected nude mouse, respectively. Relative peak areas are expressed as means ± S.D. of triplicate.
(PPTX)

S1 Table CE-MS data of 206 compounds detected from intracellular metabolite fraction of *M*. *leprae*-infected nude mouse (n = 3). (XLSX)

S2 Table CE-MS data of 49 compounds detected from intracellular metabolite fraction of uninfected nude mouse (n = 3). (XLSX)

S3 Table CE-MS data of 193 compounds determined as *M*. *leprae*-derived. Compounds also present in uninfected nude mouse (n = 35) and specifically detected from *M*. *leprae*-infected nude mouse (n = 158) were shown in shaded and plain column, respectively.
(XLSX)

S4 Table CE-MS data of 137 compounds detected from intracellular metabolite fraction of *M*. *bovis* BCG (n = 3). (XLSX)

S5 Table Comparative analyses of the relative peak areas of 84 compounds detected commonly in *M*. *leprae* (n = 3) and *M*. *bovis* BCG (n = 3). Metabolites are arranged in descending order of their mean ratio (*M*. *leprae*/*M*. *bovis* BCG).
(XLSX)
