# Supplementary material for: Profiling of Intracellular Metabolites: An Approach to Understanding the Characteristic Physiology of Mycobacterium leprae
Source: PLoS Negl Trop Dis. 2016 Aug 1;10(8):e0004881. doi: 10.1371/journal.pntd.0004881 (PMC4968842; doi:10.1371/journal.pntd.0004881)
Supplement: S1 Fig — Red and blue bar represent the relative peak areas of each metabolite detected from M. leprae-infected and uninfected nude mouse, respectively. Relative peak areas are expressed as means ± S.D. of triplicate. (PPTX) [file pntd.0004881.s001.pptx]

## Slide 1
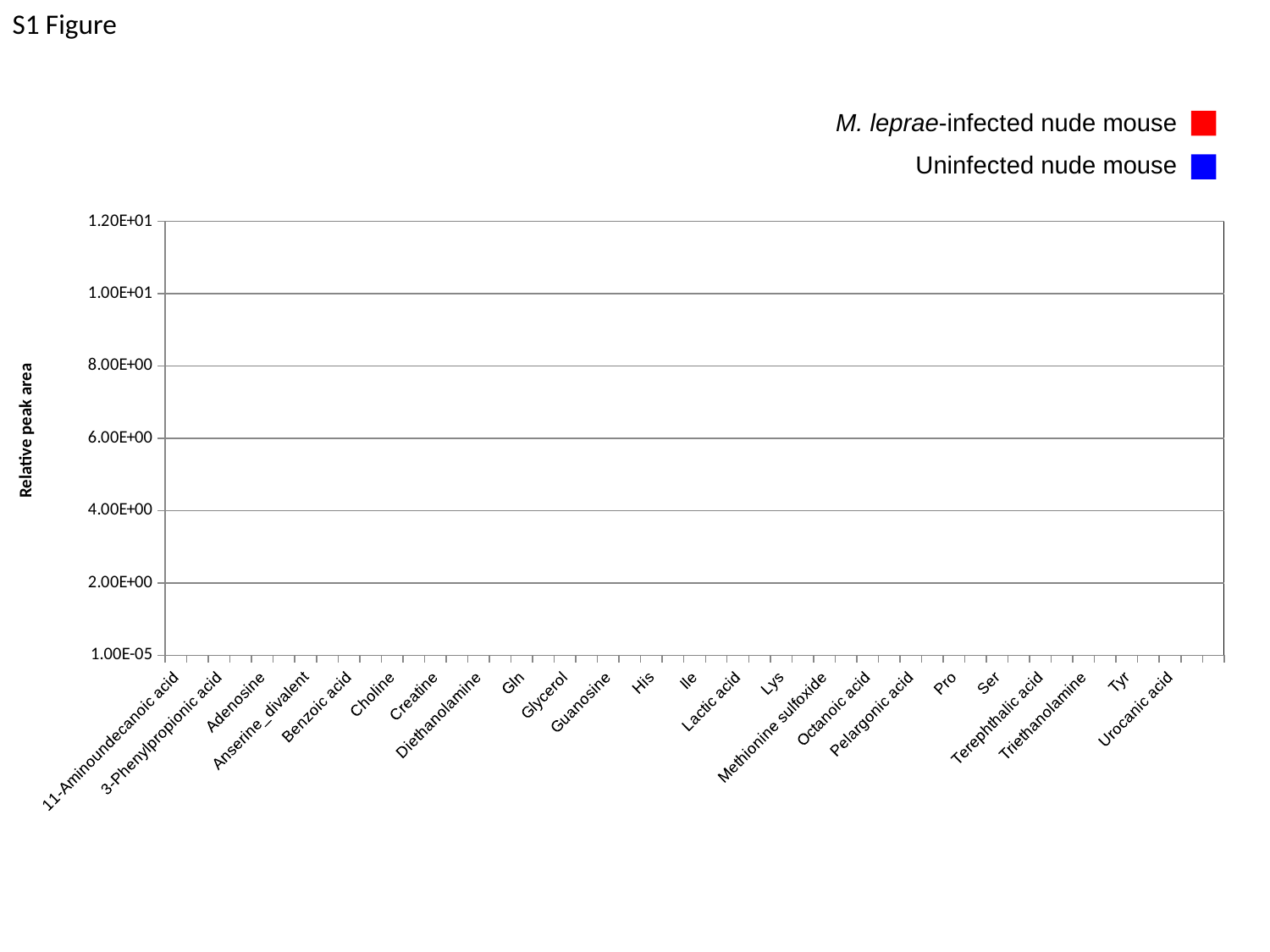

S1 Figure
M. leprae-infected nude mouse
Uninfected nude mouse
### Chart
| Category | | |
|---|---|---|
| 11-Aminoundecanoic acid | 0.0019520787333333334 | 0.001852973266666667 |
| 2-Hydroxyvaleric acid | 0.001224633437321367 | 0.001049442806757405 |
| 3-Phenylpropionic acid | 0.0019711545035836072 | 0.001737170058039917 |
| 5-Oxoproline | 0.02175941151011007 | 0.0022671321809830916 |
| Adenosine | 0.005904618266666667 | 0.00010726666666666667 |
| Ala | 0.9619312362 | 0.043923600466666664 |
| Anserine_divalent | 0.00117936 | 0.002072840266666667 |
| Arg | 1.0427776119333334 | 0.03953886306666667 |
| Benzoic acid | 0.0037166585590046298 | 0.003097770870667456 |
| Betaine | 0.1833318204666667 | 0.009948382133333334 |
| Choline | 0.1716709122 | 0.01762464346666667 |
| Citrulline | 0.0081245104 | 0.0012899096666666669 |
| Creatine | 0.23983251400000002 | 0.02918431473333333 |
| Cytidine | 0.05725452713333334 | 0.002482952 |
| Diethanolamine | 0.014847918600000001 | 0.009107553666666669 |
| Dyphylline | 0.027910460066666667 | 0.023094766133333333 |
| Gln | 0.42976608853333337 | 0.0183264162 |
| Gly | 0.5208331338000001 | 0.015591344799999999 |
| Glycerol | 1.6634962428 | 1.8076447748 |
| Glycerophosphocholine | 0.06012049453333334 | 0.0006758540666666667 |
| Guanosine | 0.0107709446 | 0.0016241378666666667 |
| Hexanoic acid | 0.0014675605504511902 | 0.0018855007995022901 |
| His | 0.33461219300000006 | 0.008161663466666667 |
| Histamine | 0.0011108708 | 0.0016429497333333334 |
| Ile | 1.0552841684666667 | 0.048159999333333335 |
| Inosine | 0.0067072762 | 0.0022766216 |
| Lactic acid | 0.15564867474886526 | 0.0424939393734892 |
| Leu | 1.8607806652666667 | 0.09469412393333333 |
| Lys | 1.2660181121333334 | 0.031846212066666664 |
| Met | 0.20352433860000005 | 0.007254069133333333 |
| Methionine sulfoxide | 0.05344364106666668 | 0.0009157372 |
| Nicotinamide | 0.010690365 | 0.0010669424666666667 |
| Octanoic acid | 0.0014199966086112317 | 0.0020259287416874217 |
| Ornithine | 0.14342823093333334 | 0.004115775666666667 |
| Pelargonic acid | 0.0031878205747302474 | 0.0035101580654261152 |
| Phe | 0.6416082974666667 | 0.029766649266666667 |
| Pro | 0.6585651730666667 | 0.023799127800000004 |
| p-Toluic acid | 0.008755424024376358 | 0.00941870373255968 |
| Ser | 0.452223023 | 0.019426086866666668 |
| Taurine | 0.17350543626666667 | 0.014704435600000001 |
| Terephthalic acid | 0.0010046015000186531 | 0.0009526372487720258 |
| Thr | 0.5745736520666668 | 0.011091910400000002 |
| Triethanolamine | 0.0008979209333333333 | 0.0006146940666666667 |
| Trp | 0.14617332293333332 | 0.0054304232 |
| Tyr | 0.4686794822 | 0.018969744733333335 |
| Urea | 0.7673063052 | 0.17237258273333333 |
| Urocanic acid | 0.0651686538 | 0.005138223666666667 |
| Val | 1.1755586923333334 | 0.056813032266666674 |Relative peak area
